# Evaluation of Pre-Harvest Nutrient Composition and Functional Active Substances in Various Lotus Roots

**DOI:** 10.3390/foods13142297

**Published:** 2024-07-21

**Authors:** Wanyu Dong, Xueting Liu, Yang Yi, Limei Wang, Wenfu Hou, Youwei Ai, Hongxun Wang, Ting Min

**Affiliations:** 1 College of Food Science & Engineering, Wuhan Polytechnic University, Wuhan 430023, China; dongwanyu0714@163.com (W.D.); lxt666662022@163.com (X.L.); yiy86@whpu.edu.cn (Y.Y.); hwf407@163.com (W.H.); aywlingyun@126.com (Y.A.); 2 Hubei Key Laboratory for Processing and Transformation of Agricultural Products, Wuhan Polytechnic University, Wuhan 430023, China; wanghongxunhust@163.com; 3 School Biology & Pharmaceutical Engineering, Wuhan Polytechnic University, Wuhan 430023, China; wanglimeiyx@163.com

**Keywords:** lotus root, variety, harvest period, nutrient composition, functional active substance

## Abstract

This study investigated the impact of variety and harvest time on the visual appearance, nutritional quality, and functional active substances of six lotus root cultivars: “Xinsanwu”, “Wuzhi No. 2”, “Baiyuzhan”, “Huaqilian”, “Elian No. 6”, and “Elian No. 5”. Samples were collected monthly from December 2023 to April 2024. A nutrient analysis revealed a decrease in the water content with a delayed harvest. The total soluble solids and soluble sugar content peaked towards the end and middle-to-late harvest periods, respectively. Starch levels initially increased before declining, while the soluble protein exhibited a triphasic trend with an initial rise, a dip, and a final increase. The vitamin C (Vc) content varied across cultivars. Functional active substances displayed dynamic changes. The total phenolics initially decreased, then increased, before ultimately declining again. The total flavonoid content varied by both cultivar and harvest time. The phenolic acid and flavonoid content mirrored the trends observed for total phenolics and total flavonoids. Gastrodin was the most abundant non-flavonoid compound across all varieties. “Wuzhi No. 2” and “Baiyuzhan” displayed higher levels of functional active substances and starch, while the Elian series and “Xinsanwu” cultivar exhibited a greater content of Vc, soluble sugar, and soluble protein. Specific harvest periods yielded optimal results: “Wuzhi No. 2” (H1 and H5), “Huaqilian” (H2), and “Baiyuzhan” (H3 and H4) demonstrated a high nutrient and functional active substance content. Overall, the lotus roots harvested in period H4 achieved the highest score. Overall, this study provides the foothold for the rapid identification of superior lotus root cultivars and the valorization of lotus root by-products via advanced processing methods. Additionally, it offers valuable insights for market participants and consumers to select optimal varieties and harvest times based on their specific needs.

## 1. Introduction

*Nelumbo nucifera*, a perennial aquatic, rhizomatous herbaceous plant, also known as lotus or “He” or “Fuqu” in Chinese, possesses an underground stem known as a “lotus root”. This ancient angiosperm originated in China and India and is a prominent aquatic vegetable in Chinese cuisine [1]. In China, the lotus is categorized into three main groups: rhizome lotus, seed lotus, and flower lotus, each with distinct characteristics. The rhizome lotus is distinguished by its tall stature, infrequent blooms, and tubers suitable for consumption. Hubei Province is a critical center for lotus cultivation, boasting a diverse collection of nearly 200 lotus root varieties, solidifying its position as a crucial region within the industry [2,3].

The lotus root, a significant export vegetable for China, boasts a rich composition of dietary fiber, starch, sugars, proteins, amino acids, minerals, vitamins, and phenolic compounds. It also holds both ornamental value and the unique characteristic of being both medicinal and edible [4,5,6]. Phenolic compounds and secondary plant metabolites offer benefits not only to the plant itself but also to human health. These compounds can contribute to the alleviation of skin disease symptoms and hinder their progression. Furthermore, consuming fruits and vegetables rich in phenolics can potentially reduce the risk of diseases mediated by oxidative stress, such as cardiovascular disease and cancer [7,8]. While current research on the lotus primarily focuses on the qualitative and quantitative analysis of phenolic compounds in lotus flowers [9,10], it should be acknowledged that lotus roots are also rich in phenolics and other functionally active substances [11]. Structurally characterized phenolic compounds identified in the lotus root include catechol, gallic acid, (+)-catechin, (−)-epicatechin, (+/−)-gallocatechin, chlorogenic acid, rutin, and more [6,12].

The quality of fruits and vegetables can vary significantly due to factors such as variety, growing period, and harvest time. These factors primarily influence the nutritional composition and functional bioactive substance content of the produce [6,13,14]. For example, a recent study [15] compared two lotus root varieties, “Elian No. 5” and “Elian No. 6”. Their evaluation of pasting and textural properties revealed that “Elian No. 5” exhibited a reduced firmness and a lower capacity to resist shear forces and thermal stress during cooking than “Elian No. 6”. Similarly, another study [13] investigated the accumulation of phenolic compounds in different parts of two pomelo varieties during their development. They found that the total phenolic content changed with the degree of maturation, initially decreasing and then increasing, reaching a peak on the 60th day. Additionally, significant differences in total phenolics were observed between the two pomelo varieties. Several studies have shown that the growing period and harvesting period also affect the flavor, processing performance, functional bioactive substance content, and antioxidant and anti-inflammatory properties of fruits and vegetables [16,17,18]. Recent research published by Wu et al. [19] analyzed the nutrient content and changes in lotus roots at different harvesting periods. They determined the optimal harvesting times as follows: September for “August-farinose”, October for “Elian No. 6”, and February for both “Elian No. 10” and “Elian No. 11”. Notably, the comprehensive nutritional quality of lotus roots harvested in March across all four varieties was found to be relatively lower. Similarly, other studies [14] evaluated the optimal harvesting period of *Dendrobium officinale*, finding that its dry matter and chemical composition differed at different harvest times, with December being the optimal harvest period. As a fruit and vegetable with significant nutritional value in China, the lotus root has been extensively studied for its nutritional components across various varieties and harvest times [6,19]. However, research on the phenolic composition and content at different varieties and harvest periods remains limited. Therefore, this study aims to conduct a systematic assessment of the nutritional components, phenolic profiles, and contents of the predominant lotus root varieties in Hubei Province during distinct harvest periods.

This study investigated six lotus root varieties: “Xinanwu”, “Wuzhi No. 2”, “Baiyuzhan”, “Huaqilian”, “Elian No. 6”, and “Elian No. 5”. The research aimed to assess the appearance (color difference, browning degree), texture, nutrient profile (total soluble solids, water content, soluble protein, soluble sugar, starch, and vitamin C), phenolic composition (total phenols, total flavonoids, and specific phenolic compounds), and antioxidant capacity (DPPH radical scavenging rate and ABTS free radical scavenging activity) across different varieties and harvest periods. This evaluation will contribute to a comprehensive scientific understanding of lotus root quality and inform strategies for optimizing both cultivation and post-harvest handling practices.

## 2. Materials and Methods

### 2.1. Materials and Instruments

Six lotus root varieties, “Xinsanwu”, “Wuzhi No. 2”, “Baiyuzhan”, “Huaqilian”, “Elian No. 6”, and “Elian No. 5”, were harvested at different harvest periods (H1–H5) from 16 December 2023, to 16 April 2024, in Hankou North. Samples were collected monthly, with one sample per period (H1, H2, H3, H4, H5). The corresponding temperature ranges for each harvest period were as follows: H1 (1–11 °C), H2 (3–5 °C), H3 (2–12 °C), H4 (8–15 °C), and H5 (12–20 °C). Following harvest, the lotus roots were promptly transported to the laboratory for a 24 h pre-cooling period at 4 °C.

Analytical grade chemicals, including anhydrous ethanol, sodium nitrate, sodium hydroxide, aluminum nitrate, anhydrous sodium carbonate, glucose, concentrated sulfuric acid, anhydrous disodium hydrogen phosphate, anhydrous disodium dihydrogen phosphate, glacial acetic acid, and 1,1-diphenyl-2-picrylhydrazyl, were purchased from Sinopharm Chemical Reagent Co., Ltd. (Shanghai, China) Folin–Ciocalteu’s phenol was obtained from Wuhan Feiyang Bio-tech Co., Ltd. (Shanghai, China) High purity methanol, acetonitrile, and glacial acetic acid (≥99.9%, chromatographic grade) were purchased from Sigma-Aldrich. Standards (≥98%) including coumarin, pyrogallol, (+)-catechin, gastrodin, hyperin, rutin, quercetin, and hydroquinone were obtained from Aladdin Reagent (Shanghai) Co., Ltd. (Shanghai, China) Additional standards (≥98%) including quercetin, (−)-epicatechin, chlorogenic acid, and (+/−)-gallocatechin were purchased from Shanghai Yuanye Bio-technology Co., Ltd. (Shanghai, China) Assay kits for total starch, vitamin C, soluble protein, and total antioxidant capacity (ABTS method) were obtained from Beijing Solarbio Science & Technology Co., Ltd. (Beijing, China) Nanjing Jiancheng Bioengineering Institute, and Beyotime Biotechnology (Shanghai, China), respectively. All unspecified reagents were of analytical grade.

The experimental setup utilized various instruments: a EOS 550D digital camera for image capture (Canon Co., Ltd., Beijing, China); a low-temperature refrigerator (Sanyo, Japan); a JZ-500 general colorimeter manufactured (Shenzhen Jinhuai Instrument Equipment Co., Ltd., Shenzhen, China); an IMS-20 ice-making machine (Changshu Xueke Electric Appliance Co., Ltd., Changshu, China); an XHF-D high-speed disperser and an ultrasonic cleaner(Ningbo Xinzhi Biotechnology Co., Ltd., Ningbo, China); an A360 UV-Vis spectrophotometer (Aoyi Instruments Co., Ltd., Shanghai, China); a high-speed refrigerated centrifuge (Shanghai Anting Scientific Instrument Factory, Shanghai, China); a multifunctional enzyme-linked immunosorbent assay (ELISA) reader (PerkinElmer, Waltham, MA, USA); an HPLC 1269 II system(Agilent Technologies Co., Ltd., Beijing, China); a texture analyzer (Shanghai Bao Sheng Industrial Development Co., Ltd., Shanghai, China); and an HH-8 digital display constant temperature water bath (Jintan District Bai Ta Xinbao, Jintan, China).

### 2.2. Methods

#### 2.2.1. Sample Pretreatment

To prepare the lotus root samples, surface mud was removed with tap water. The roots were then sectioned using sterilized knives, ensuring complete cuts at the nodes to minimize air exposure to the internal flesh.

Samples were selected from lotus roots harvested at different periods (H1, H2, H3, H4, H5). To account for potential variations in material condition across harvesting times, a preliminary treatment process was applied. This involved peeling and dicing the samples, followed by rapid freezing using liquid nitrogen. The frozen samples were then stored at −80 °C in an ultra-low-temperature freezer for further analysis.

#### 2.2.2. Appearance

The assessment of lotus root appearance quality was conducted using a digital camera (Canon EOS 550D) to capture standardized photographs of the whole lotus root.

#### 2.2.3. Color Difference

In accordance with the methodology described in the literature [20], a representative area of the lotus root skin was randomly chosen, and its edible segment’s surface color parameters (L value, a value, and b value) were measured at five different points using a JZ-500 general colorimeter. The color difference (ΔE) was calculated using the following equation:

(1) $$\Delta E=\sqrt{{\left(L^{*}-L_{0}^{*}\right)}^{2}+{\left(a^{*}-a_{0}^{*}\right)}^{2}+{\left(b^{*}-b_{0}^{*}\right)}^{2}}$$

where L_0_, a_0_, and b_0_ were all values on the H1 period, and L*, a*, and b* were readings at each sampling point during the harvest period.

#### 2.2.4. Water Content

The water content was determined following the methodology outlined in the literature [21], with some modifications: A tray was placed in a 105 °C drying box and weighed to a constant weight (±2 mg). Then, two slices of lotus root were weighed and placed into the tray and dried in a 105 °C drying box to a constant weight (±2 mg). The water content was expressed in % after three repetitions for each sample.

#### 2.2.5. Browning Degree

The browning degree was evaluated following the procedure described in the literature [22]. Briefly, at 4 °C, 30 mL distilled water was mix with a 3.0 g sample, homogenized, and then centrifuged for 5 min at 10,000× *g*. The supernatant in the centrifuge tube was collected. The absorbance is measured at 410 nm after zeroing the spectrophotometer with distilled water; this measurement was repeated three times. The results are then expressed as A410 × 10.

#### 2.2.6. Total Soluble Solids

The total soluble solids content was determined using a portable refractometer [23]. To prepare the sample, 10 g of tissue was ground in a mortar with an ice bath. The homogenate was then transferred to a centrifuge tube and centrifuged at 10,000 rpm for 5 min. The supernatant was measured thrice on the prism of the refractometer to obtain the reading.

#### 2.2.7. Texture

The texture analysis followed the method reported in the literature [23]. Lotus root flesh was cut into cubes measuring approximately 1 cm on each side (1 cm × 1 cm × 1 cm). A texture analyzer was set to TPA mode with a P/45 probe, a 100 g trigger force, and the following speeds: 10.0 mm/s for initial speed, 0.5 mm/s for compression speed, and 10.0 mm/s for end ascending speed. A 5 s dwell time was set between two compressions, and the maximum deformation was set to 35%. The experiment was conducted with 10 biological replicates.

#### 2.2.8. Total Phenolic Content

The total phenolic content of the fresh-cut lotus root was determined using the Folin–Ciocalteu method [24]. Briefly, a 3.0 g sample of flesh tissue was homogenized with 30 mL 60% ethanol and centrifuged, and the absorbance was triply measured at 760 nm after the reaction with the Folin–Ciocalteu reagent. A standard curve prepared with gallic acid was used to calibrate the results, expressed as milligrams of gallic acid equivalents (GAE) per 100 g.

#### 2.2.9. Total Flavonoid Content

The total flavonoid content was determined using the method described in the literature [25]. Five grams of flesh tissue and 50 mL of 60% ethanol were used to prepare the extraction mixture, which was centrifuged for 10 min (4 °C, 10,000× *g*). Following sample preparation, the absorbance was triply measured at a wavelength of 510 nm. Rutin was employed as a standard for quantification, and the total flavonoid content was expressed as milligrams of rutin per 100 g (mg rutin/100 g).

#### 2.2.10. Soluble Sugar

The soluble sugar content was determined as described in the literature [26] with few modifications. Absorbance was measured at 485 nm after three repetitions for each sample. A standard curve was prepared using an anhydrous glucose solution, and the mass fraction of soluble sugar was then calculated using the following formula:

(2) $$\mathrm{Soluble}\;\mathrm{sugar}\;\mathrm{mass}\;\mathrm{fraction}\;(\%)=\frac{m^{\prime }\times V\times N}{V_{S}\times m\times 10^{6}}\times 100$$

where m′ represents the mass of the soluble sugar determined from the standard curve, μg; V denotes the complete volume of the extracted sample, mL; N signifies the dilution ratio of the extracted sample; V_s_ is the volume of the sample extract used for the measurement, mL; and m is the weight of the sample, g.

#### 2.2.11. Starch Content

The starch content was determined following the manufacturer’s instructions for the total starch assay kit obtained from Beijing Solarbio Science & Technology Co., Ltd. A total of 3 biological replicates were established.

#### 2.2.12. Soluble Protein

The soluble protein content was determined following the manufacturer’s instructions for the commercially available soluble protein assay kit obtained from Nanjing Jiancheng Bioengineering Institute. A total of 4 g of flesh tissue and 36 mL of phosphate buffered solution (0.1 mol/L, pH = 7.0) were used to prepare the extraction mixture, which was centrifuged for 10 min (4 °C, 10,000× *g*); then, the supernatant was saved for subsequent measurement. Absorbance was measured three times at 595 nm, and the results were expressed in mg/g.

#### 2.2.13. Vitamin C Content

The vitamin C content was determined according to the instructions of the kit (Nanjing Jiancheng Bioengineering Institute). The sample was homogenized with phosphate buffered solution (0.1 mol/L, pH = 7.0) at a ratio of 1:9 under ice bath conditions. After centrifugation at 4 °C at 10,000× *g* for 10 min, the supernatant was saved for later use. Absorbance was measured three times for each sample at 536 nm, and the results were expressed in μg/g.

#### 2.2.14. DPPH Radical Scavenging Rate

The DPPH radical scavenging rate was determined according to the method described in the literature [27]. A total of 2 g of the sample was homogenized in 25 mL ethanol and sonicated (50 °C) for 30 min. After centrifuging for 10 min (4 °C, 10,000× *g*), the supernatant was diluted ten times. Absorbance was triply measured for each sample at 517 nm, with anhydrous ethanol used to blank the instrument. The results were expressed as a percentage (%).

#### 2.2.15. ABTS Radical Scavenging Ability

The ABTS radical scavenging ability was measured following the manufacturer’s instructions for the total antioxidant capacity assay kit. A total of 5 g of the sample was weighted, homogenized in 25 mL phosphate buffered solution (0.1 mol/L, pH = 7.0) in ice mortar, and centrifuged for 10 min (4 °C, 10,000× *g*). The absorbance of each sample was measured at 734 nm with three biological replicates and the results were expressed as Trolox equivalent antioxidant capacity (mM Trolox/g).

#### 2.2.16. Monomeric Phenol Content

The lotus root phenolic extraction method was conducted as described in the literature [6], with few modifications. Briefly, 32 g of the ground lotus root sample was homogenized with 160 mL of the pre-cooled 40% ethanol solution adjusted to pH 3.0. The mixture was then ultrasonicated for 72 min followed by centrifugation at 4500 rpm for 10 min. The supernatant was collected by filtration, and the residue was re-extracted with 200 mL of pre-cooled 40% ethanol (pH 3.0) using ultrasonication for 10 min and centrifugation. The combined supernatants were concentrated using vacuum rotary evaporation and re-dissolved in methanol to a final volume of 10 mL. A high-performance liquid chromatography (HPLC) analysis was performed using two separate programs for the optimal detection of different phenolic compounds. The first program, designed for pyrogallol, gastrodin, coumarin, (+/−)-gallocatechin, hydroquinone, (+)-catechin, chlorogenic acid, (−)-epicatechin, and quercetin, utilized a mobile phase consisting of solvent A (methanol) and solvent B (0.4% glacial acetic acid) at a flow rate of 1.0 mL/min. The column temperature was maintained at 30 °C with UV detection at 280 nm. An injection volume of 20 μL was used with a linear elution program: 0–40 min (5–25% A), 40–50 min (25–50% A), 50–65 min (50–70% A), 65–66 min (70–100% A), 66–72 min (100% A), 72–73 min (100–5% A), and 73–80 min (5% A). The second program, designed for quercetin, rutin, and hyperin, employed a mobile phase composed of solvent A (acetonitrile) and solvent B (0.4% glacial acetic acid) at a flow rate of 1.0 mL/min with a column temperature of 30 °C and UV detection at 280 nm. The injection volume remained at 20 μL, and the linear elution program was as follows: 0–10 min (5–25% A), 10–20 min (25–35% A), 20–21 min (35–100% A), 21–25 min (100% A), 25–26 min (100–5% A), and 26–30 min (5% A). The sample was measured thrice.

### 2.3. Statistical Analysis

Data were processed, analyzed, and visualized using Microsoft Excel (version 2019, Microsoft Corporation, Redmond, WA, USA) and Origin software (version 2021, OriginLab Corporation, Northampton, MA, USA). Statistical significance testing, correlation analysis, and principal component analysis were performed using SPSS (version 20.0, IBM Corporation, Armonk, NY, USA).

## 3. Results and Discussion

### 3.1. Nutritional Components and Functional Active Substances

#### 3.1.1. Appearance

**Figure A1:**
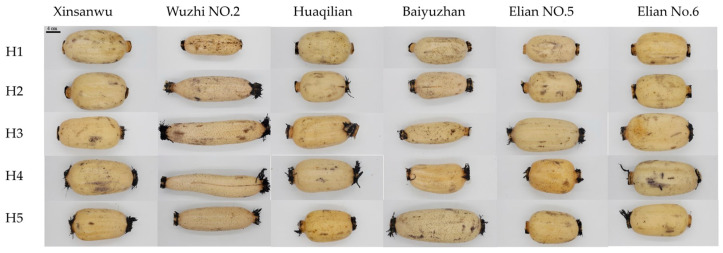
Appearance difference of various lotus roots at different harvest times.

Table 1 illustrates the visual differences in lotus root appearance across varieties and harvest times. “Wuzhi No. 2” and “Baiyuzhan” exhibited a slender oval shape, while the remaining four varieties possessed larger and more rounded nodes (Figure A1). Table 1 presents the L* values, indicating lightness, of all varieties. All L* values were above 56 (*p* < 0.05), but generally showed a downward trend throughout the harvest period. Notably, “Baiyuzhan” and “Wuzhi No. 2” maintained a higher and less pronounced decrease in L* values compared to “Elian No. 5” and “No. 6”, whose L* values experienced a significant decline. The a* and b* values, representing redness–greenness and yellowness–blueness, respectively, generally exhibited an upward trend across all varieties during the harvest period. “Wuzhi No. 2” and “Baiyuzhan” possessed higher a* values, suggesting a redder skin color compared to other varieties. Conversely, “Elian No. 5” and “No. 6” exhibited significantly higher b* values, indicating a tendency towards a more yellowish skin color. The ΔE values of lotus roots across all varieties exhibited a general upward trend with the extension of the harvest period, and distinctions could be discerned among different harvest periods (*p* < 0.05). This trend was corroborated by the visual appearance of the intact lotus roots as depicted in photographs taken throughout the various harvest periods.

#### 3.1.2. Texture

Table 2 reveals a consistent upward trend in hardness, chewiness, and cohesiveness for all lotus root varieties as the harvest period progresses. Significant differences (*p* < 0.05) were observed in these textural properties within the same variety at different harvest times. However, the cohesiveness between different varieties at the same harvest time did not exhibit significant differences (*p* > 0.05). Previous research [28] suggests that rising temperatures lead to the expansion of water volume within fruits and vegetables. This expansion, in turn, promotes increased cell strength, ultimately contributing to greater hardness at later harvest stages. Similarly, a recent study [29] demonstrated that a decrease in inherent water content within the lotus root leads to increased cohesiveness between tissues, resulting in enhanced hardness. These findings align with the observations of this study. Hardness is critical in determining the commercial quality, storability, and shelf life of fruits and vegetables. Many studies [30,31] have established a positive correlation between hardness and storage performance. Therefore, the observed increase in hardness with extended harvest periods suggests potentially improved storability for lotus roots. Cohesiveness refers to the degree of tight binding between internal components, while chewiness represents the energy required to chew food to a swallowable state [32,33]. As the harvest period progresses, the textural data suggest a strengthening of the internal bonds within all lotus root varieties, consequently increasing the energy needed for mastication.

#### 3.1.3. Browning Degree, Total Soluble Solids, Water Content

**Figure 1:**
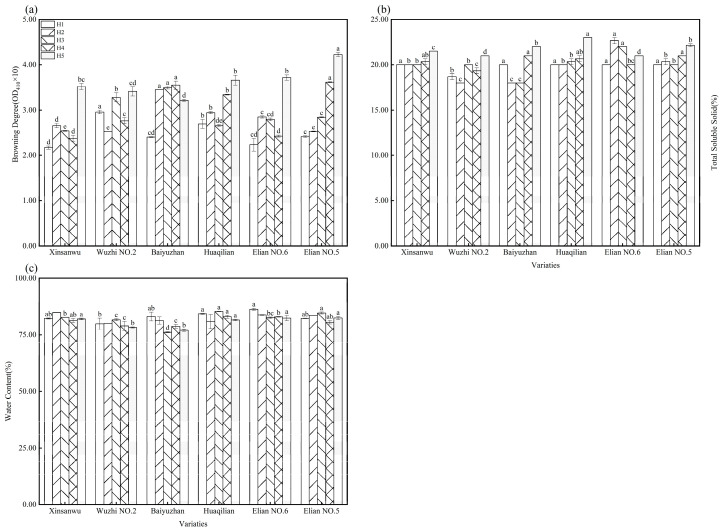
Browning degree, soluble solids, and water content of lotus root in different varieties and different harvesting periods. (**a**) Browning degree; (**b**) total soluble solid; and (**c**) water content; different letters, such as a, b, c, etc., indicate significant differences among different varieties during the same harvest time (*p* < 0.05).

The degree of browning in freshly-cut produce significantly influences consumer purchasing decisions, making it a crucial quality metric [34]. As shown in Figure 1a, browning increases with delayed harvest. By harvest period H5 (April), the browning degree at the center of the lotus root exhibited a significant rise, particularly in varieties Elian No. 5 and No. 6, reaching 75.52% and 66.82%, respectively. Soluble solids, essential nutrients in fruits and vegetables, serve as a key indicator of lotus root quality [35]. Figure 1b reveals that “Wuzhi No. 2” and “Baiyuzhan” consistently possessed lower soluble solids than other varieties throughout the harvest period. However, all varieties exhibited the highest soluble solids content by harvest period H5 (April). This finding aligns with recent research [36], which reported that crop cycle, harvest month, and variety could affect the soluble solids content in cucumbers. Similarly, another research study [37] reported increasing soluble solids content with ripening in “Orin” apples, which is consistent with our observations. As shown in Figure 1c, the water content of all varieties tended to decrease with delayed harvest, with the highest water content observed during harvest period H1 (December). “Wuzhi No. 2” and “Baiyuzhan” showed a significantly lower water content than other varieties throughout the harvest period. In daily practice, water content is an experience-based indicator for measuring lotus root crispness; a higher water content suggests crispiness, while a lower water content indicates a powdery texture [15]. Therefore, “Wuzhi No. 2” and “Baiyuzhan” can be considered more powdery than other varieties. The data also suggest that water content changes can influence the texture within the same lotus root variety as the harvest period extends.

#### 3.1.4. Soluble Sugar, Starch, Soluble Protein, and Vc Content

**Figure 2:**
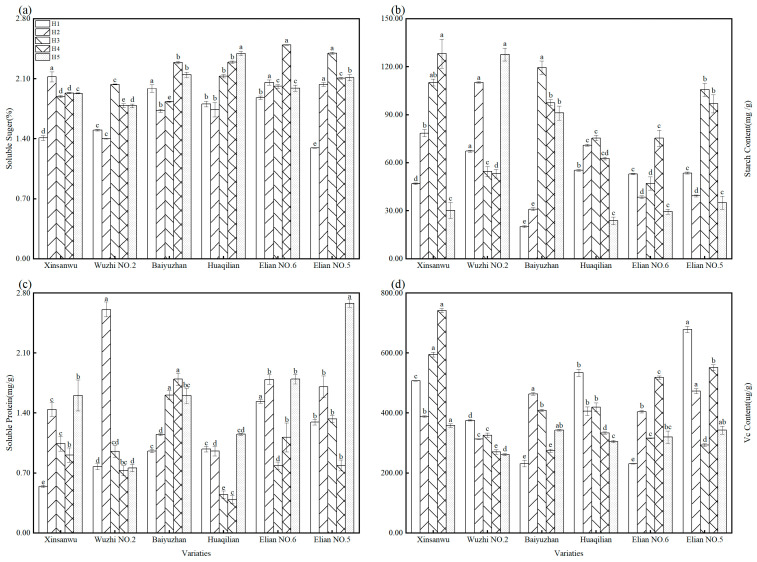
Content of soluble sugar, starch, soluble protein, and Vc of lotus root in different varieties and different harvesting periods. (**a**) Soluble sugar; (**b**) starch content; (**c**) soluble protein; and (**d**) Vc content; different letters, such as a, b, c, etc., indicate significant differences among different varieties during the same harvest time (*p* < 0.05).

Figure 2a illustrates the soluble sugar content across various lotus root varieties throughout the harvest period. A consistent pattern emerged, with all varieties exhibiting the lowest content initially. The content then increased, peaked in the middle to late stages, and subsequently decreased. This peak ranged from 1.29% to 2.50%. The greatest difference in soluble sugar content between varieties occurred during harvest period H1. “Baiyuzhan” boasted a content 1.53 times higher than “Elian No. 5.” However, “Elian No. 5” demonstrated the most significant change in soluble sugar content as the harvest progressed. Its content reached a high of 2.40% in period H3 (February), representing a 1.85-fold increase compared to H1. Seasonal temperature fluctuations likely contributed to this observed pattern. Previous studies have shown that decreasing temperatures can lead to the accumulation of soluble sugars [38], accounting for the peak in soluble sugar content observed during the middle of the harvest period.

Figure 2b reveals significant differences (*p* < 0.05) in starch content across lotus root varieties at various harvest times. The data demonstrated a general trend of initial increase followed by a subsequent decrease, with the starch content ranging from 20.18 to 119.43 mg/g. Notably, “Wuzhi No. 2”, “Xinsanwu”, and “Baiyuzhan” consistently exhibited a higher starch content compared to other varieties. These findings were consistent with recent research [39], which reported a range of a 1.3% to 13.7% starch content across 10 different lotus root varieties from various growing regions. Previous studies suggest seasonal variations in the carbohydrate composition of plant tissues, with the peak starch content occurring in autumn and the lowest levels observed in winter [40]. During this period, starch undergoes a conversion into soluble sugars. Conversely, early spring witnesses the reconversion of these soluble sugars back into starch. However, multiple factors influence this starch conversion process, including fruit weight, soluble solids content, flesh firmness, and variations in absorbance [41].

Figure 2c illustrates the range of soluble protein content in lotus root varieties, varying from 0.39 to 2.58 mg/g. A recent study [42] investigated the yield and quality of seven lotus root varieties, reporting a soluble protein content range of 0.7 to 3.3 mg/g. This observed difference might be attributed to variations in both the specific varieties studied and the sampling period employed. The data for all varieties, except for “Baiyuzhan”, revealed a recurring pattern of a low point in the soluble protein content during harvest periods H3 and H4, followed by an increase, then a decrease, and finally another increase. In contrast, “Baiyuzhan” maintained a consistently higher level of soluble protein content throughout the harvest period.

As shown in Figure 2d, the Vc content in lotus root varieties ranged from 231.28 to 741.96 μg/g. Interestingly, the Vc content patterns differed between varieties. While “Baiyuzhan” and “Elian No. 6” demonstrated an initial rise followed by a decline, others exhibited a decrease at the beginning of the harvest period. Notably, “Xinsanwu” and “Elian No. 5” maintained a relatively stable and high Vc content throughout. Similar trends in vitamin C content variation have been observed in other studies. Li et al. [43] reported that the vitamin C content in edible peonies peaked at stage S2, exhibiting a rise-and-fall pattern. This aligned to some extent with the observed trend in “Baiyuzhan” and “Elian No. 6.” However, the influence of various factors on vitamin C content should be borne in mind, including plant maturity, variety, temperature, and environment [44]. These factors contribute to the observed variations in Vc content change across the six lotus root varieties throughout the harvest period.

#### 3.1.5. Phenolic Compounds and Antioxidant Ability

**Figure 3:**
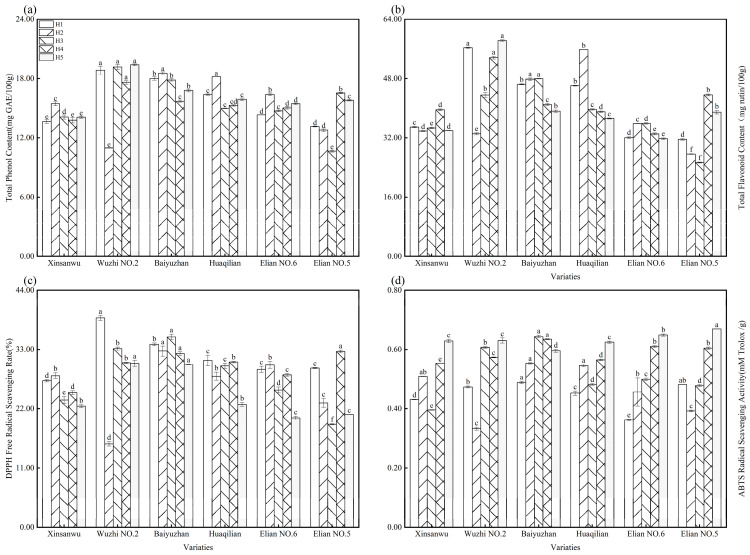
Total phenol and total flavonoid content; DPPH free radical scavenging rate and ABTS free radical scavenging activity of lotus root in different varieties and different harvesting periods. (**a**) Total phenol; (**b**) total flavonoid; (**c**) DPPH free radical scavenging rate; and (**d**) ABTS free radical scavenging activity; different letters, such as a, b, c, etc., indicate significant differences among different varieties during the same harvest time (*p* < 0.05).

Figure 3a presents the total phenolic content (TPC) of lotus root varieties, ranging from 10.65 to 19.39 mg GAE/100 g. Across all six varieties, the TPC exhibited a general trend of increasing followed by a decrease, and then another increase as the harvest period progresses. This pattern aligned broadly with the findings of Mijin et al. [45] regarding TPC variations in jackfruit at different harvest times. Notably, “Xinsanwu”, “Baiyuzhan”, “Huaqilian”, and “Elian No. 6” reached their peak TPC during period H2 (January). Additionally, “Wuzhi No. 2” and “Baiyuzhan” consistently exhibited a significantly higher TPC compared to other varieties (*p* < 0.05). Previous research has investigated the influence of temperature and light on phenolic compound production in cranberry fruits and leaves. These studies suggest that cooler temperatures and light exposure promote the biosynthesis of these compounds [46]. This finding may explain the observed peak in TPC for lotus root during period H2 in this study, which likely coincides with cooler temperatures and potentially greater light exposure.

Figure 3b depicts the variations in total flavonoid content (TFC) across lotus root varieties throughout the harvest period. The data revealed distinct patterns between varieties. “Baiyuzhan”, “Huaqilian”, and “Elian No. 6” exhibited an initial increase in TFC followed by a subsequent decrease. Conversely, “Xinsanwu”, “Elian No. 5”, and “Wuzhi No. 2” demonstrated an initial decline in TFC, followed by an increase, and finally another decrease. The total flavonoid content of lotus root ranged from 25.33 to 58.26 mg rutin/100 g. Notably, “Wuzhi No. 2” maintained a consistently high level of total flavonoids throughout the harvest period, with the exception of period H2. A recent study [47] investigated changes in flavonoid compounds within dried tangerine peel at different harvest times. Their findings revealed an increasing and then decreasing trend in total flavonoid content as the harvest period progressed, which aligned generally with the observations in this experiment for some varieties.

Lotus root is rich in phenolic compounds, known for their potent antioxidant properties and efficient free radical scavenging abilities [48]. Prior research [49] has established a significant positive correlation between the antioxidant capacity of plant materials and their phenolic content. The DPPH (2,2-diphenyl-1-picrylhydrazyl) free radical scavenging rate and ABTS (2,2′-azino-bis(3-ethylbenzothiazoline-6-sulfonic acid)) radical scavenging activity serve as valuable indicators for evaluating the antioxidant capacity of lotus root, reflecting the anti-free radical activity of phenolic compounds [50]. Figure 3c illustrates the DPPH free radical scavenging rates of the six lotus root varieties, exhibiting a general trend of decrease followed by an increase and, finally, another decrease. The scavenging rates ranged from 15.48% to 38.84%. Notably, “Wuzhi No. 2” and “Baiyuzhan” consistently maintained a higher level of DPPH activity throughout the harvest period. Additionally, the DPPH scavenging rates for most varieties reached their lowest point during period H5. This observed trend aligned broadly with the pattern of total phenolic content depicted in Figure 3a. Figure 3d presents the ABTS free radical scavenging activity of all lotus root varieties, demonstrating a generally increasing trend as the harvest period progressed. The activity ranged from 0.33 to 0.67 Mm Trolox/g. This pattern deviated slightly from the trends observed for both total phenolic content and DPPH scavenging rate. However, it aligns with a past study [16], which reported an increase in antioxidant capacity with increasing ripeness. Conversely, another study [51] observed a decreasing trend in DPPH and ABTS scavenging rates for different pomelo varieties with extended harvest periods. Research on other produce, such as cashews, has shown an increase in antioxidant capacity with ripeness [52].

#### 3.1.6. Monomeric Phenolic

Table 3 details the changes in individual phenolic compound content across the six lotus root varieties at various harvest times. Chlorogenic acid was the predominant phenolic acid identified in all varieties, followed by coumarin. The content of these phenolic acids exhibited a recurring pattern of decrease, increase, and then another decrease, mirroring the trend observed for total phenolic content (Figure 3a). Notably, peak levels of phenolic acids were generally reached during the middle and late harvest periods. “Wuzhi No. 2” demonstrated a significantly higher content of chlorogenic acid compared to other varieties throughout the harvest period (*p* < 0.05), reaching a maximum of 2.27 μg/g during period H5. The primary flavonoids detected in the lotus root were (+/−)-gallocatechin and (+)-catechin. “Elian No. 6” exhibited the highest content of (+/−)-gallocatechin at 55.44 μg/g during period H4, which was 2.4 times the content found in “Huaqilian” (23.07 μg/g) for the same period. (−)-Epicatechin, while exhibiting a high content, was only detected in some varieties during periods H3 and H4. The trend observed for flavonoid content change aligned generally with that of total flavonoids (Figure 3b). Similarly, “Wuzhi No. 2” and “Baiyuzhan” maintained consistently high levels of flavonoids throughout the harvest period. Gastrodin, a non-flavonoid substance, was the most abundant phenolic compound detected across all varieties and at all harvest times. During period H5, “Huaqilian” exhibited the highest gastrodin content, reaching 191.31 μg/g, which was 2.29 times greater than the content observed in “Baiyuzhan” (83.70 μg/g) for the same period. Similar to other phenolic substances, the gastrodin content in all varieties peaked during the middle and late harvest periods. Hydroquinone was primarily detected during periods H3 to H5, exhibiting a relatively low content and also reaching its peak later in the harvest period. The statistical analysis revealed significant differences (*p* < 0.05) in the content of phenolic substances in lotus roots at different harvest times. Similar observations have been reported for wheat, where variety and maturity stage significantly impact the phenolic content [53]. Additionally, Fernandes et al. [54] documented changes in the flavonoid content of calendula with an extended harvest period. Various environmental factors, including relative humidity, temperature, and light intensity, can influence the content of phenolic substances [55]. The content of the main phenolic acids (e.g., chlorogenic acid), flavonoids ((+/−)-gallocatechin), and non-flavonoids (e.g., gastrodin) in the lotus root varied throughout the harvest period. Each substance generally peaked during the middle and late harvest periods. This trend might be attributable to lower temperatures, which are typically experienced during these later stages.

### 3.2. Principal Component Analysis

Principal component analysis (PCA) has emerged as a valuable tool for quality assessment in agricultural products, as evidenced by its application to winter jujubes and broccoli [56,57]. PCA streamlines the assessment process by reducing the dimensionality of data and eliminating redundant information from multiple sources. This simplification leads to faster and more accurate evaluations than individual assessments of each quality metric. Furthermore, PCA effectively addresses potential biases arising from intercorrelations among traits, which could otherwise skew assessment outcomes [58]. In this study, an integrative assessment model was constructed to quantitatively evaluate the quality attributes of lotus roots across various cultivars and harvest periods. By employing PCA, ten quality metrics—water content, total soluble solids, soluble sugars, starch, soluble protein, Vc, total phenolic content, total flavonoid content, DPPH radical scavenging capacity, and ABTS radical scavenging activity—were condensed into four principal components (Table 4). This approach facilitated a more comprehensive evaluation of the lotus root’s overall quality profile. The eigenvalues of the first four principal components, all exceeding 1, were 3.269, 2.106, 1.440, and 1.174, respectively. These components collectively accounted for 79.89% of the total variance in the data.

The first principal component (PC_1_) primarily captured the variation associated with active components, such as total phenols, total flavonoids, and the DPPH free radical scavenging rate. This suggests a strong positive correlation between these factors. The second principal component (PC_2_) was mainly associated with soluble solids and soluble sugars, indicating a potential link between these two variables. The third principal component (PC_3_) focused on indicators related to the lotus root’s physiological state, such as water content, starch content, and soluble protein content. These factors likely covary and influence aspects of lotus root texture and composition. Finally, the fourth principal component (PC_4_) primarily reflected the content of Vc. By utilizing the comprehensive factor scores obtained from the PCA, the following Formulas (3)–(7) were derived for further data processing (Table 5):

PC_1_ = −0.354X_1_ − 0.140X_2_ − 0.105X_3_ + 0.090X_4_ − 0.242X_5_ − 0.060X_6_ + 0.497X_7_ + 0.514X_8_ + 0.444X_9_ + 0.254X_10_ (3)

PC_2_ = −0.004X_1_ + 0.522X_2_ + 0.519X_3_ − 0.249X_4_ + 0.123X_5_ − 0.360X_6_ + 0.175X_7_ − 0.042X_8_ − 0.061X_9_ + 0.464X_10_ (4)

PC_3_ = −0.561X_1_ − 0.093X_2_ − 0.032X_3_ + 0.510X_4_ + 0.491X_5_ − 0.056X_6_ − 0.143X_7_ − 0.010X_8_ − 0.298X_9_ + 0.248X_10_ (5)

PC_4_ = 0.025X_1_ + 0.192X_2_ + 0.389X_3_ + 0.477X_4_ − 0.449X_5_ + 0.556X_6_ − 0.132X_7_ − 0.106X_8_ + 0.061X_9_ + 0.203X_10_ (6)

PC = 0.409PC_1_ + 0.264PC_2_ + 0.180PC_3_ + 0.147PC_4_ (7)

The principal components likely represent the following: PC_1_, PC_2_, PC_3_, and PC_4_ correspond to the first, second, third, and fourth principal components, respectively. There is no “composite principal component” denoted by “PC” in standard PCA analysis. Data for various nutritional and functional indicators of the lotus root (water content, total soluble solids, soluble sugars, starch, soluble protein, Vc, total phenols, total flavonoids, DPPH free radical scavenging rate, and ABTS radical scavenging activity) were standardized as X_1_ through X_10_.

Table 5 reveals that lotus roots from harvest period H4 generally scored higher in the first and third principal components compared to other months. This observation could be attributed to its higher content of both common functional active substances and starch. In the second principal component, the H5 lotus root exhibited the highest scores, indicating a higher carbohydrate content during this harvest period. The fourth principal component highlighted a higher Vc content in the H4 lotus root compared to other periods. The comprehensive scores identified “Wuzhi No. 2” harvested in H1 and H5, “Huaqilian” harvested in H2, and “Baiyuzhan” harvested in H3 and H4 as having superior nutritional value and a functional active substance content. Notably, the overall highest comprehensive score belonged to the H4 lotus root, suggesting a relatively higher concentration of both nutritional components and functional active substances during this harvest period.

## 4. Conclusions

This study investigated the nutritional composition and functional active substance content of six lotus root varieties across different harvest periods. Significant variations were observed between varieties and harvest times. The L value and ΔE of lotus roots from different varieties generally showed an upward trend as the harvest period extended, while the trends of a and b values for different varieties did not align. Meanwhile, textural indicators exhibited an increasing trend with the extension of the harvest period. “Wuzhi No. 2” and “Baiyuzhan” exhibited a higher phenolic and starch content. Additionally, their lower water content translated into a more powdery and waxy texture, making them well-suited for soups and lotus root starch production. Conversely, the two “Elian” series varieties, with their higher water content, vitamin C content, and crisper texture, were found to be more suitable for stir-frying after purchase. The principal component analysis identified “Wuzhi No. 2” harvested in H1 and H5, “Huaqilian” harvested in H2, and “Baiyuzhan” harvested in H3 and H4 as having a superior nutritional and functional active substance content. Notably, the overall highest score belonged to the lotus root harvested during H4. This suggests a peak in the nutritional and functional active substance content during the H4 harvest period compared to others. However, it is important to acknowledge that H4 also coincided with peak hardness and browning in the lotus root, which might negatively impact consumer preferences despite the higher nutritional value. This study provides valuable insights into the development of advanced lotus root processing techniques and the differentiation of varieties for the marketplace. Consumers and the industry can leverage these findings to make informed choices regarding variety selection and optimal harvest times based on specific needs. Future research can explore targeted product development by strategically selecting varieties and harvest times to address diverse consumer demands and enrich the variety of lotus root products available in the market.

## Figures and Tables

**Table 1:** Color difference of various lotus roots of different harvest time.

		Xinsanwu	Wuzhi No. 2	Baiyuzhan	Huaqilian	Elian No. 6	Elian No. 5
	H1	61.59 ± 0.48 Acd	61.25 ± 0.40 d	62.80 ± 0.24 Abc	61.64 ± 0.77 Acd	64.54 ± 0.41 Aa	63.12 ± 0.25 Ab
	H2	61.25 ± 0.47 A	61.58 ± 0.62	60.86 ± 0.37 AB	61.53 ± 0.57 A	62.06 ± 0.24 AB	62.87 ± 0.63 AB
L*	H3	59.77 ± 0.52 AB	60.88 ± 0.67	60.34 ± 0.80 B	59.61 ± 0.77 B	60.63 ± 0.90 B	59.82 ± 0.96 BC
	H4	60.38 ± 0.59 ABab	59.35 ± 1.30 b	61.71 ± 0.37 ABa	59.21 ± 0.18 Bb	59.95 ± 0.45 Bab	59.80 ± 0.33 Cb
	H5	58.13 ± 1.02 B	59.11 ± 2.12	59.97 ± 1.07 B	56.32 ± 0.52 C	59.34 ± 1.61 B	57.31 ± 1.63 C
	H1	6.89 ± 0.41 BCb	8.94 ± 0.60 Ba	6.38 ± 0.08 Cbc	5.45 ± 0.17 Cc	7.15 ± 0.21 Ab	7.15 ± 0.13 Bb
	H2	6.05 ± 0.24 Cbc	11.42 ± 0.24 Aa	6.35 ± 0.23 Cbc	5.39 ± 0.27 Cd	5.79 ± 0.06 Bcd	6.50 ± 0.15 Bb
a*	H3	7.12 ± 0.04 BCc	8.28 ± 0.37 Bb	9.60 ± 0.27 Aa	7.72 ± 0.16 Abc	7.59 ± 0.44 Abc	6.97 ± 0.29 Bc
	H4	7.20 ± 0.30 Bbc	8.05 ± 0.23 Bb	9.60 ± 0.39 Aa	6.49 ± 0.26 Bc	5.47 ± 0.14 Bd	9.98 ± 0.45 Aa
	H5	9.29 ± 0.53 Aa	8.15 ± 0.45 Bab	7.82 ± 0.47 Bb	8.25 ± 0.15 Aab	7.64 ± 0.33 Ab	9.20 ± 0.46 Aa
	H1	18.66 ± 0.62 bc	19.26 ± 0.19 Ab	15.03 ± 0.46 Dd	17.23 ± 0.88 BCc	21.06 ± 0.53 Aa	15.274 ± 0.36 Cd
	H2	18.15 ± 0.43 b	19.68 ± 0.57 Aa	14.52 ± 0.21 Dc	15.39 ± 0.18 Cc	19.46 ± 0.18 Ba	17.69 ± 0.47 Bb
b*	H3	17.36 ± 0.62 c	18.29 ± 1.03 Abc	18.87 ± 0.94 Babc	19.98 ± 0.78 Aab	20.73 ± 0.42 Aa	19.10 ± 0.46 ABabc
	H4	19.47 ± 1.21 ab	16.13 ± 0.61 Bcd	20.92 ± 0.53 Aa	18.38 ± 0.81 ABbc	15.19 ± 0.28 Cd	21.15 ± 0.51 Aa
	H5	20.27 ± 1.52 a	14.28 ± 0.88 Bb	17.13 ± 0.30 Cab	19.81 ± 1.11 Aa	20.03 ± 0.40 ABa	20.07 ± 1.12 Aa
	H1	0.00	0.00	0.00	0.00	0.00	0.00
	H2	1.61 ± 0.26 BCc	2.99 ± 0.39 Bab	2.11 ± 0.33 Cbc	2.25 ± 0.10 Cbc	3.29 ± 0.14 Ba	2.91 ± 0.29 Bab
ΔE	H3	2.50 ± 0.53 Bc	2.70 ± 0.40 Bc	6.07 ± 0.39 Aa	4.62 ± 0.29 Bab	4.16 ± 0.77 Bbc	5.36 ± 0.59 Bab
	H4	3.04 ± 0.34 Bb	4.25 ± 0.96 Bb	6.90 ± 0.43 Aa	3.24 ± 0.40 Cb	7.67 ± 0.37 Aa	8.24 ± 0.98 Aa
	H5	5.28 ± 1.19 Aab	6.98 ± 0.78 Aab	4.21 ± 0.73 Bb	6.79 ± 0.74 Aab	5.38 ± 1.47 AB	8.39 ± 1.18 Aa

Different letters such as A, B, C, etc. indicate significant differences among harvest times of the same variety (*p* < 0.05); different letters such as a, b, c, etc. indicate significant differences among different varieties during the same harvest time (*p* < 0.05).

**Table 2:** Texture difference of various lotus roots of different harvest time.

Texture		Xinsanwu	Wuzhi No. 2	Baiyuzhan	Huaqilian	Elian No. 6	Elian No. 5
Hardness gf	H1	7358.45 ± 366.26 Bd	8372.77 ± 283.99 Bb	8043.64 ± 247.68 Bbc	8370.05 ± 230.89 BCb	9692.01 ± 376.90 ABa	7523.43 ± 233.66 BCc
	H2	7541.97 ± 358.40 Bab	8056.18 ± 215.10 Ba	6518.07 ± 322.20 Cb	8241.09 ± 168.31 Ca	8379.51 ± 780.27 Ba	7571.44 ± 390.56 BCab
	H3	8937.18 ± 219.36 Aabc	8336.39 ± 279.05 Bc	8838.48 ± 391.04 Babc	9756.13 ± 481.81 Aa	9562.44 ± 114.01 ABab	8642.21 ± 248.31 ABbc
	H4	9196.05 ± 324.95 Ab	9089.24 ± 303.37 ABb	10,981.51 ± 526.29 Aa	9587.17 ± 614.85 ABab	9755.39 ± 69,904 ABab	9077.23 ± 688.25 Ab
	H5	9675.20 ± 298.20 Aab	9945.14 ± 484.92 Aab	9091.88 ± 348.72 Bb	9985.85 ± 433.59 Aab	10,646.76 ± 458.33 Aa	9140.80 ± 565.08 Ab
Chewiness gf	H1	6687.66 ± 168.03 Cb	6717.01 ± 122.24 Bb	5234.35 ± 91.07 Cc	6359.36 ± 166.22 bC	7100.94 ± 344.11 Bbc	7536.34 ± 382.31 Ba
	H2	6716.30 ± 449.06 Cabc	7369.20 ± 372.37 Bab	5265.04 ± 334.85 Cc	8193.31 ± 523.68 Ba	7104.23 ± 559.62 Bab	6037.34 ± 305.49 Cbc
	H3	7861.87 ± 501.90 BCbc	7004.27 ± 211.44 Bc	7629.86 ± 466.89 Bbc	8587.70 ± 305.01 ABab	9094.54 ± 491.58 ABa	7298.40 ± 185.06 BCc
	H4	8761.98 ± 436.42 ABa	8906.39 ± 750.36 Aa	9903.25 ± 360.07 Aa	917.07 ± 187.47 ABa	9095.33 ± 872.02 ABa	7869.63 ± 571.18 Bb
	H5	9759.31 ± 346.54 A	9277.92 ± 413.72 A	9239.16 ± 317.12 A	9569.81 ± 631.84 A	9821.65 ± 826.41 A	9675.80 ± 451.33 A
Cohesiveness	H1	0.74 ± 0.03 Cb	0.78 ± 0.01 Cb	0.55 ± 0.01 Dc	0.75 ± 0.02 Cb	0.75 ± 0.03 Cb	0.88 ± 0.02 Ba
	H2	0.86 ± 0.03 B	0.87 ± 0.05 AB	0.81 ± 0.02 C	0.90 ± 0.04 B	0.86 ± 0.03 B	0.86 ± 0.04 B
	H3	0.88 ± 0.04 B	0.84 ± 0.02 BC	0.89 ± 0.03 BC	0.89 ± 0.05 B	0.95 ± 0.05 AB	0.85 ± 0.03 B
	H4	0.95 ± 0.03 AB	0.92 ± 0.02 AB	0.97 ± 0.04 AB	0.97 ± 0.05 AB	0.90 ± 0.04 B	0.92 ± 0.03 B
	H5	0.98 ± 0.02 A	0.94 ± 0.02 A	1.01 ± 0.05 A	1.03 ± 0.03 A	1.02 ± 0.04 A	1.04 ± 0.03 A

Different letters such as A, B, C, etc. indicate significant differences among harvest times of the same variety (*p* < 0.05); different letters, such as a, b, c, etc., indicate significant differences among different varieties during the same harvest time (*p* < 0.05).

**Table 3:** Changes in the monomeric phenolic content of Nelumbo nucifera of various lotus roots at different harvest times. (nd means undetected; content unit: μg/g).

	Phenols		Xinsanwu	Wuzhi No. 2	Baiyuzhan	Huaqilian	Elian No. 6	Elian No. 5
Phenolic Acids	Quercetin	H1	0.45 ± 0.01 Cc	0.49 ± 0.01 Da	nd	nd	nd	0.47 ± 0.01 Bb
		H2	nd	nd	nd	nd	nd	nd
		H3	0.60 ± 0.01 Bc	0.83 ± 0.01 Ab	0.78 ± 0.05 Ab	0.57 ± 0.01 Bc	1.07 ± 0.03 Aa	nd
		H4	0.86 ± 0.01 Aa	0.69 ± 0.01 Bb	0.62 ± 0.01 Bc	0.54 ± 0.01 Cd	0.52 ± 0.01 Cd	0.47 ± 0.01 Be
		H5	0.79 ± 0.07 Aa	0.62 ± 0.01 Cbc	0.60 ± 0.01 Cc	0.77 ± 0.01 Aa	0.72 ± 0.01 Bab	0.71 ± 0.01 Aab
	Coumarin	H1	1.00 ± 0.02 Cab	0.94 ± 0.01 Dab	1.05 ± 0.06 Cab	1.35 ± 0.32 Ba	0.86 ± 0.01 Cab	0.66 ± 0.04 Cb
		H2	0.86 ± 0.02 Dbc	0.95 ± 0.17 Dcb	0.95 ± 0.01 Dab	0.88 ± 0.02 Bbc	1.04 ± 0.02 Ca	0.76 ± 0.02 Cd
		H3	1.17 ± 0.07 Bd	1.92 ± 0.01 Bb	2.07 ± 0.01 Aa	0.84 ± 0.06 Be	1.24 ± 0.01 Cd	1.71 ± 0.04 Bc
		H4	2.00 ± 0.02 Ab	1.54 ± 0.01 Cbc	1.77 ± 0.02 Bbc	1.41 ± 0.03 Bd	3.24 ± 0.27 Aa	2.91 ± 0.25 Aa
		H5	1.92 ± 0.06 Ac	2.74 ± 0.02 Aa	1.71 ± 0.02 Bcd	2.69 ± 0.21 Aa	2.30 ± 0.02 Bb	1.52 ± 0.02 Bd
	Chlorogenic Acid	H1	nd	1.48 ± 0.35 Ba	0.90 ± 0.00 c	1.02 ± 0.05 BCb	nd	nd
		H2	1.21 ± 0.11	1.28 ± 0.10 C	nd	1.06 ± 0.08	1.18 ± 0.05 B	1.21 ± 0.19
		H3	1.09 ± 0.04 c	1.61 ± 0.06 Bb	1.82 ± 0.07 Aa	0.90 ± 0.02 Cd	1.13 ± 0.01 Bc	1.15 ± 0.02 c
		H4	1.20 ± 0.03 b	1.38 ± 0.03 BCab	1.60 ± 0.16 Aa	1.27 ± 0.02 Bb	1.13 ± 0.10 Bb	1.35 ± 0.07 ab
		H5	1.07 ± 0.06 c	2.27 ± 0.02 Aa	1.11 ± 0.01 Bc	1.76 ± 0.20 Ab	1.81 ± 0.17 Ab	1.25 ± 0.16 c
Flavonoids	Quercetin	H1	nd	0.32 ± 0.01 Bc	0.24 ± 0.01 Cd	nd	0.45 ± 0.01 b	0.52 ± 0.03 Ba
		H2	nd	nd	nd	nd	0.31 ± 0.01 B	nd
		H3	0.30 ± 0.01 Bc	0.41 ± 0.01 Aa	0.30 ± 0.01 Bc	nd	0.36 ± 0.01 Ab	0.37 ± 0.01 Cb
		H4	0.35 ± 0.02 ABb	nd	0.37 ± 0.03 Ab	nd	0.28 ± 0.01 Cc	0.54 ± 0.01 Aa
		H5	0.35 ± 0.01 Ac	0.44 ± 0.01 Aa	0.41 ± 0.01 Aabc	0.42 ± 0.04 ab	0.37 ± 0.01 Abc	0.41 ± 0.02 Cabc
	Rutin	H1	0.63 ± 0.04 Ecd	0.68 ± 0.06 Ebc	1.08 ± 0.06 Da	0.52 ± 0.13 Dd	0.78 ± 0.03 Db	0.59 ± 0.03 Ecd
		H2	1.18 ± 0.08 Da	1.27 ± 0.10 Da	0.75 ± 0.07 Eb	1.15 ± 0.03 Ba	1.17 ± 0.06 Ca	1.16 ± 0.02 Da
		H3	1.38 ± 0.01 Cd	1.55 ± 0.01 Cc	2.52 ± 0.12 Ba	0.83 ± 0.01 Cf	1.66 ± 0.02 Bb	1.27 ± 0.01 Ce
		H4	1.47 ± 0.01 Bd	2.23 ± 0.01 Ab	2.71 ± 0.05 Aa	1.09 ± 0.01 Be	1.63 ± 0.01 Bc	1.45 ± 0.01 Bd
		H5	1.74 ± 0.04 Ae	1.67 ± 0.04 Be	2.21 ± 0.01 Cb	2.07 ± 0.03 Ac	2.43 ± 0.02 Aa	1.97 ± 0.01 Ad
	Hyperin	H1	0.26 ± 0.11 Dbc	0.21 ± 0.01 Ecd	0.35 ± 0.06 Db	0.51 ± 0.01 Ca	0.24 ± 0.01 Ecd	0.15 ± 0.03 Ed
		H2	0.42 ± 0.02 Cb	0.50 ± 0.01 Da	0.23 ± 0.01 Ed	0.30 ± 0.01 Dc	0.32 ± 0.02 Dc	0.42 ± 0.01 Db
		H3	0.79 ± 0.01 Bd	1.13 ± 0.01 Cb	1.75 ± 0.02 Aa	0.60 ± 0.01 Ce	1.16 ± 0.01 Bb	0.85 ± 0.01 Cc
		H4	0.94 ± 0.01 Ad	1.20 ± 0.02 Bc	0.93 ± 0.02 Bd	0.83 ± 0.01 Be	1.40 ± 0.01 Ab	1.62 ± 0.02 Aa
		H5	0.28 ± 0.02 De	1.56 ± 0.01 Aa	0.85 ± 0.01 Cc	1.25 ± 0.07 Ab	0.72 ± 0.01 Cd	1.22 ± 0.01 Bb
	(+)-catechin	H1	14.41 ± 1.34 Cc	18.71 ± 0.32 Db	21.78 ± 1.49 Da	21.04 ± 0.95 Bab	10.39 ± 0.17 Dd	4.95 ± 0.17 De
		H2	22.00 ± 1.92 Aa	13.03 ± 0.27 Ed	18.50 ± 0.39 Db	15.12 ± 0.36 Dc	18.68 ± 1.26 Bb	14.10 ± 0.45 Ccd
		H3	18.49 ± 0.28 Bc	33.91 ± 2.01 Bb	46.98 ± 1.66 Aa	13.50 ± 0.18 Ed	16.25 ± 0.05 Ccd	13.74 ± 0.01 Cd
		H4	21.85 ± 0.04 Ade	29.46 ± 0.14 Ca	27.96 ± 1.65 Cab	18.69 ± 0.15 Ce	23.56 ± 1.18 Acd	25.23 ± 1.50 Abc
		H5	16.66 ± 0.06 Bd	41.79 ± 2.12 Aa	33.62 ± 0.15 Bb	32.02 ± 0.32 Ab	25.00 ± 0.27 Ac	19.06 ± 1.05 Bd
	(−)-Epicatechin	H1	nd	4.54 ± 0.22 B	nd	nd	nd	nd
		H2	nd	nd	nd	nd	nd	nd
		H3	4.20 ± 0.16 c	6.78 ± 0.17 Ab	11.01 ± 0.20 a	nd	4.28 ± 0.04 c	nd
		H4	3.50 ± 0.13 c	4.65 ± 0.30 Bb	5.43 ± 0.06 a	nd	4.32 ± 0.09 b	nd
		H5	nd	nd	nd	nd	nd	nd
	(+/−)-Gallo-Cate-chin	H1	20.01 ± 0.79 Cc	49.20 ± 1.36 Aa	28.01 ± 0.10 Eb	28.63 ± 2.47 Bb	11.18 ± 0.16 Ed	8.23 ± 1.61 De
		H2	42.93 ± 1.23 Ab	19.55 ± 3.17 De	28.72 ± 0.57 Dd	46.09 ± 0.13 Aa	34.60 ± 1.83 Cc	28.51 ± 1.59 Bd
		H3	19.61 ± 0.19 Cd	34.95 ± 0.77 Bb	53.31 ± 0.30 Aa	12.84 ± 3.02 Ce	23.71 ± 0.19 Dc	18.12 ± 0.20 Cd
		H4	26.72 ± 0.04 Bd	26.37 ± 0.84 Cd	39.89 ± 0.10 Cc	23.07 ± 1.21 Bd	55.44 ± 2.75 Aa	45.21 ± 1.87 Ab
		H5	29.30 ± 2.03 Bb	49.04 ± 0.75 Aa	45.73 ± 0.19 Ba	52.32 ± 5.29 Aa	48.38 ± 0.35 Ba	31.05 ± 0.28 Bb
Non-Flav-ono-ids	Gastrodin	H1	108.68 ± 3.62 Cbc	155.81 ± 40.64 Aa	48.35 ± 6.01 Cd	72.24 ± 8.58 Bcd	127.90 ± 3.51 Cab	77.80 ± 1.08 Dcd
		H2	73.28 ± 2.19 Dc	71.27 ± 1.57 Cc	58.82 ± 4.37 Cd	88.33 ± 3.62 bB	83.99 ± 3.72 Db	102.04 ± 4.27 Ca
		H3	127.76 ± 0.78 Bb	112.67 ± 0.58 Bc	148.71 ± 5.71 Aa	89.73 ± 2.90 Bd	57.07 ± 4.87 Ee	49.66 ± 1.80 Ee
		H4	133.48 ± 5.36 ABc	90.04 ± 0.72 BCd	147.25 ± 3.38 Ab	91.27 ± 1.08 Bd	151.33 ± 0.78 Bab	159.40 ± 3.81 Aa
		H5	138.23 ± 2.54 Ac	120.27 ± 4.84 Bd	83.70 ± 3.03 Be	191.31 ± 7.68 Aa	166.00 ± 6.47 Ab	122.75 ± 3.85 Bcd
	Hydroquinone	H1	nd	0.49 ± 0.04 D	nd	0.75 ± 0.01 B	nd	nd
		H2	nd	nd	nd	nd	nd	nd
		H3	0.79 ± 0.06 Ab	0.62 ± 0.02 Cc	1.30 ± 0.06 a	0.57 ± 0.02 Bc	0.63 ± 0.02 Cc	0.64 ± 0.03 Bc
		H4	0.78 ± 0.01 Ad	0.87 ± 0.03 Bc	1.23 ± 0.01 a	0.65 ± 0.01 Be	1.07 ± 0.01 Ab	1.26 ± 0.02 Aa
		H5	0.65 ± 0.01 Bc	1.46 ± 0.04 Aa	1.30 ± 0.40 ab	1.26 ± 0.06 Aab	0.90 ± 0.03 Bbc	0.70 ± 0.03 Bc

Different letters such as A, B, C, etc. indicate significant differences among harvest times of the same variety (*p* < 0.05); different letters such as a, b, c, etc. indicate significant differences among different varieties during the same harvest time (*p* < 0.05).

**Table 4:** Principal component variance characteristic values, contribution rates, and load matrix values.

Variable	PC_1_	PC_2_	PC_3_	PC_4_
Water Content	−0.641	−0.007	−0.673	0.027
Total Soluble Solid	−0.253	0.758	−0.111	0.208
Soluble Sugar	−0.190	0.752	−0.038	0.422
Starch Content	0.162	−0.361	0.612	0.516
Soluble Protein	−0.437	0.178	0.590	−0.486
Vc Content	−0.108	−0.523	−0.067	0.602
Total Phenol Content	0.899	0.253	−0.171	−0.143
Total Flavonoid Content	0.930	−0.060	−0.012	−0.115
DPPH	0.803	−0.089	−0.357	0.067
ABTS	0.460	0.673	0.298	0.220
Characteristic Values	3.269	2.106	1.440	1.174
Variance Percentage/%	32.694	21.056	14.396	11.737
Accumulate/%	32.694	53.750	68.147	79.883

**Table 5:** Principal component scores, composite scores, and ranking of component indicators of different varieties of lotus root at different harvesting periods.

Varieties	PC_1_	PC_2_	PC_3_	PC_4_	PC	Rank
Xin-H1	0.313	0.161	−0.374	0.531	0.181	26
Wu-H1	1.316	0.225	−0.269	0.314	0.595	9
Bai-H1	0.797	0.821	−0.564	0.171	0.466	16
Hua-H1	0.581	0.376	−0.449	0.591	0.343	23
6-H1	0.044	0.513	−0.429	0.188	0.104	28
5-H1	0.230	0.073	−0.182	0.618	0.171	27
Xin-H2	0.264	0.669	−0.169	0.629	0.347	22
Wu-H2	−0.172	−0.104	0.672	0.067	0.033	30
Bai-H2	0.977	0.425	−0.299	0.292	0.501	14
Hua-H2	1.006	0.561	−0.101	0.486	0.613	8
6-H2	0.245	0.957	−0.354	0.432	0.352	21
5-H2	−0.196	0.518	−0.215	0.454	0.084	29
Xin-H3	0.171	0.159	−0.005	0.929	0.247	24
Wu-H3	0.957	0.890	−0.255	0.500	0.654	7
Bai-H3	1.261	0.436	0.549	0.625	0.822	2
Hua-H3	0.417	0.579	−0.469	0.837	0.361	20
6-H3	0.287	0.896	−0.316	0.566	0.380	19
5-H3	−0.308	0.683	0.121	0.813	0.195	25
Xin-H4	0.434	0.254	0.183	1.329	0.473	15
Wu-H4	1.115	0.662	−0.105	0.328	0.660	6
Bai-H4	0.727	1.062	0.359	0.657	0.739	4
Hua-H4	0.563	0.893	−0.367	0.808	0.518	11
6-H4	0.361	0.860	−0.090	1.006	0.506	13
5-H4	0.825	0.706	−0.028	1.058	0.674	5
Xin-H5	0.194	1.038	−0.038	0.417	0.407	18
Wu-H5	1.365	0.778	0.273	0.695	0.915	1
Bai-H5	0.744	1.037	0.338	0.688	0.740	3
Hua-H5	0.333	1.450	−0.215	0.587	0.566	10
6-H5	0.190	1.114	0.010	0.329	0.422	17
5-H5	0.214	1.332	0.196	0.275	0.514	12

H1–H5 represented Dec. 2023–Apr. 2024, “Xin” means “Xinsanwu”, “Wu” means “Wuzhi No. 2”, “Bai” means “Baiyuzhan”, “Hua” means “Huaqilian”, “6” means “Elian No. 6”, and “5” means “Elian No. 5”.

## Data Availability

The original contributions presented in the study are included in the article; further inquiries can be directed to the corresponding author.
